# Health and Economic Impact of COVID-19 Surveillance Testing in Seattle Homeless Shelters: A Cost-Effectiveness Analysis

**DOI:** 10.1016/j.focus.2024.100307

**Published:** 2024-12-05

**Authors:** Sarah N. Cox, Eric J. Chow, Melissa A. Rolfes, Emily Mosites, Monisha Sharma, Helen Y. Chu, Marita Zimmermann

**Affiliations:** 1 Department of Epidemiology, School of Public Health, University of Washington, Seattle, Washington; 2 Department of Medicine, University of Washington, Seattle, Washington; 3 Public Health – Seattle & King County, Seattle, Washington; 4 Centers for Disease Control and Prevention, Atlanta, Georgia; 5 Department of Global Health, University of Washington, Seattle, Washington; 6 Department of Pharmacy, School of Pharmacy, University of Washington, Seattle, Washington; 7 Institute for Disease Modeling, Bill & Melinda Gates Foundation, Seattle, Washington

**Keywords:** Homeless shelters, surveillance, COVID-19, cost-utility, cost-effective, vaccination

## Abstract

**Introduction:**

COVID-19 surveillance in congregate settings is important to mitigating disease, but the health and economic impact of testing remains unclear.

**Methods:**

The authors developed a Markov model to project the cost-utility of COVID-19 testing strategies in homeless shelters from the healthcare payer and societal perspective over 1 year. Model inputs utilized data from residents aged ≥18 years across 23 Seattle shelters from January 1, 2020, to May 31, 2021. No in-shelter surveillance was compared with scenarios of 2 COVID-19 testing strategies implemented monthly: polymerase chain reaction (PCR) testing and rapid antigen testing; scenarios in which only PCR testing was available were also evaluated. The primary health outcome was quality-adjusted life years. Interventions were considered cost-effective if the incremental cost-effectiveness ratio was ≤$150,000 per quality-adjusted life year and dominant if they saved costs and provided health effects.

**Results:**

When assuming the availability of both antigen and PCR tests, most rapid antigen testing strategies were cost-effective, whereas PCR testing was dominated by antigen testing. Compared with no in-shelter surveillance, antigen testing increased mean quality-adjusted life years by 0.0009 (0.03% infections averted) at an incremental cost of $97/resident from the healthcare perspective (incremental cost-effectiveness ratio=$112,352/quality-adjusted life year gained) and $8/resident from the societal perspective (incremental cost-effectiveness ratio=$9,627/quality-adjusted life year gained) at 75% vaccination coverage. PCR testing was not cost-effective when antigen testing was available but was cost-effective compared with no surveillance at low vaccination coverage levels (<30% coverage from the healthcare perspective and ≤48% coverage from the societal perspective). Probabilistic sensitivity analysis showed that antigen testing was cost-effective in 62% and 86% of simulations from the healthcare and societal perspectives, respectively.

**Conclusions:**

Modeled findings show that COVID-19 testing in shelters can be a cost-effective pandemic response. Antigen testing remained cost-effective at high vaccination levels, whereas PCR testing was most effective at low vaccination levels if antigen testing was not available.

## HIGHLIGHTS

• COVID-19 rapid antigen testing is likely to be cost-effective in homeless shelters.
• Without COVID-19 antigen testing, PCR testing is optimal at low vaccination coverage levels.
• Modeled findings help decision-makers quantify the value of shelter surveillance.

## INTRODUCTION

The coronavirus disease 2019 (COVID-19) pandemic has caused enormous morbidity and mortality with disparate impacts across socioeconomic and racial groups in the U.S.^1^^,^^2^ Among people experiencing homelessness, the risk of death from COVID-19 is estimated to be 30%–50% higher than among the overall population.^3^^,^^4^ Of an estimated 582,462 people experiencing homelessness each evening in the U.S., approximately 60% reside in homeless shelters.^5^ Ensuring the inclusion of shelters in COVID-19 surveillance efforts is paramount because people experiencing sheltered homelessness face an increased risk of respiratory viral infections due to the challenges of overcrowding, maintaining physical distance, poor ventilation, and sharing of hygiene facilities.^6^, ^7^, ^8^, ^9^ Previous data show that a greater proportion of sheltered people experiencing homelessness tested positive for both current and prior severe acute respiratory syndrome coronavirus 2 (SARS-CoV-2) infections compared with those experiencing unsheltered homelessness, suggesting a higher risk of transmission in shelters.^10^ Therefore, surveillance of COVID-19 incidence and vaccination, particularly in congregate settings and among marginalized populations, is important to mitigating pandemic harms.

A community-based surveillance study of respiratory viruses in Seattle shelters found an unmet need for routine COVID-19 testing outside of clinical settings for people experiencing homelessness to identify outbreaks and prevent further transmission.^11^^,^^12^ Increasing access to COVID-19 testing and enhancing mitigation strategies for COVID-19 among people experiencing homelessness are a public health priority. However, little is known about the health impact and economic impact of COVID-19 surveillance testing and vaccination programs in homeless shelters.

This study aimed to estimate the health and economic impact of pandemic COVID-19 surveillance testing for adults across Seattle King County shelters. Findings can support recommendations for COVID-19 and future outbreak mitigation for key stakeholders, including shelters, public health, and policymakers.

## METHODS

The authors developed a Markov model to project health outcomes and costs and estimate the cost-utility of monthly COVID-19 surveillance testing strategies in homeless shelters. This economic evaluation was the second step in the analysis plan (Appendix Figure 1, available online) and followed the Consolidated Health Economic Evaluation Reporting Standards guideline.^13^

### Scenarios

A baseline scenario of no in-shelter testing was compared with 2 intervention scenarios in which shelter management actively encouraged all residents to participate in COVID-19 testing once per month regardless of symptoms with polymerase chain reaction (PCR) testing (Scenario 1) and rapid antigen (Ag) testing (Scenario 2). Scenarios were also evaluated in which it was assumed that Ag testing was not available (i.e., comparing the availability of PCR testing only with no surveillance). Monthly testing was modeled because this was the most realistic testing frequency among shelter residents in the Seattle Flu Study (SFS). Although testing frequency varied throughout the pandemic and changed on the basis of individual preference—and select individuals opted for more frequent testing (which may be more ideal)—monthly testing was more commonly observed.^14^ Both PCR and Ag tests had different sensitivity and specificity for those who were symptomatic versus asymptomatic. These represent different testing strategies employed over the study period that could be adopted more widely, such as in other congregate living facilities.

### Model

**Figure 1:**
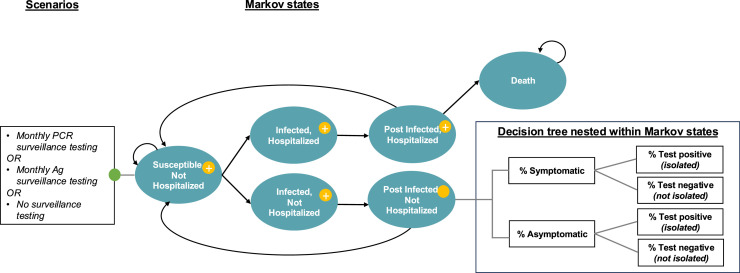
Markov model to estimate health outcomes and costs and determine the cost-utility of monthly surveillance testing strategies in homeless shelters. All Markov states except death were repeated for vaccinated and unvaccinated proportions of the population (leading to 11 distinct Markov states). At each time step, those who were susceptible faced a probability of becoming infected and not hospitalized, becoming infected and hospitalized, or remaining susceptible. Hospitalized represents COVID-19 hospitalizations only (not hospitalized represents COVID-19 non-hospitalizations only). Those who were infected and hospitalized remained in a tunnel state for 2 time steps and experienced a probability of dying or returning to the susceptible state after ≥3 months. All infected and not hospitalized individuals in the model returned to the susceptible state after ≥3 months. As demonstrated in nested decision trees (represented by yellow circles), all scenarios across the model assumed that a proportion of the shelter population was either symptomatic or asymptomatic, and test sensitivity and specificity were based on type of test used. All hospitalized COVID-19 cases were assumed to be symptomatic and remain symptomatic for 2 time steps. Test performance impacted a shelter residents’ likelihood of testing positive or negative for COVID-19 and thus whether they were placed in isolation (e.g., put in a separate room or moved to an isolation and quarantine facility). To account for the differences in PCR versus Ag testing scenarios and reduced transmission due to isolation, each probability of infection was multiplied by (1− the proportion isolated in the previous cycle) as a proxy to incorporate dynamic incidence in the model.

Figure 1 depicts the 11-state Markov model used to project the baseline (i.e., no surveillance testing) and intervention strategies of monthly PCR surveillance testing and Ag surveillance testing. Costs and outcomes were modeled over a 1-year time horizon with a monthly time cycle.

At the start of the model time horizon, the population was assumed to be in 1 of 2 susceptible, not hospitalized states: one with individuals vaccinated against COVID-19 (≥1 dose in the past year) and the second with individuals unvaccinated against COVID-19. Vaccination coverage remained constant for the duration of the time horizon, with 75% of the population having received ≥1 dose and 25% having had 0 doses of COVID-19 vaccine in the past year.^15^ In addition, all transition probabilities were differentiated by vaccination status.

At each monthly time step, those susceptible faced a probability of becoming infected and not hospitalized, becoming infected and hospitalized, or remaining susceptible. Those who were infected and hospitalized remained in a tunnel state and experienced a probability of dying or returning to the susceptible state after ≥3 months. All infected and not hospitalized individuals in the model returned to the susceptible state after ≥3 months to allow for the possibility of reinfection.^16^^,^^17^ In each model state, health outcomes and costs accrued proportionally on the basis of the disease duration. For instance, hospitalization costs and health outcomes only accumulated for the number of days a person was hospitalized (e.g., 4 days). Similarly, nonhospitalized individuals accrued costs and reductions in health outcomes only for the number of days they were symptomatic within the infected and not-hospitalized state. For the remainder of the state, they accumulated no costs or health outcomes, ensuring accurate representation of short events within longer time cycles.

As demonstrated in the nested decision tree for each Markov state except for death, a proportion of the population was assumed to be either symptomatic or asymptomatic, and test sensitivity and specificity were assumed based on the test type used (Table 1^14^^,^^15^^,^^18^, ^19^, ^20^, ^21^, ^22^, ^23^, ^24^, ^25^, ^26^, ^27^, ^28^, ^29^, ^30^, ^31^, ^32^, ^33^, ^34^, ^35^ and Figure 1). All hospitalized COVID-19 cases were assumed to be symptomatic and remain symptomatic for 2 time steps. Test performance impacted shelter residents’ likelihood of testing positive or negative for COVID-19 and thus whether or not they were placed in isolation (e.g., put in a separate room within the shelter or moved to an isolation and quarantine facility outside of the shelter).^36^ To account for the differences in PCR versus Ag testing scenarios and reduced transmission due to isolation, each probability of infection was multiplied by (1− the proportion isolated in the previous cycle) as a proxy to incorporate dynamic incidence in the model.

**Table 1:** Model Inputs

Parameters	Mean or median	SE or range	Distribution	Source
Costs (2023 USD)				
COVID-19 PCR test	$19.56	$17.60–$21.52	Normal	SFS expert opinion^18^
Operational cost per COVID-19 PCR test	$18.66	$16.70–$20.62	Normal	SFS expert opinion^18^
COVID-19 rapid Ag test	$5.50	$4.52–$6.48	Normal	SFS expert opinion^18^
Operational cost per COVID-19 Ag test	$4.98	$4.00–$5.96	Normal	SFS expert opinion^18^
Over the counter medications, daily^a^	$0.54	$0.15–$0.93	Gamma	Bartsch et al. (2021)^19^
Outpatient care, daily^b^	$152.54	$130.20–$174.88	Normal	CMS^20^
Hospitalization non-ICU, daily	$1,804.27	$1,196.79–$2,411.75	Normal	HHS^19^^,^^21^
Hospitalization ICU, daily	$6,867.27	$6,066.88–$7,667.66	Normal	HHS^19^^,^^21^
Annual wages (all occupations)	$52,376.61	$30,921.54–$124,737.99	Normal	Bureau of Labor Statistics^22^
Utility weights				
Healthy QALY, 18–64 years	0.92	0.04	Normal	Gold et al. (1998)^23^
Healthy QALY, ≥65 years	0.84	0.04	Normal	Gold et al. (1998)^23^
Mild nonspecific symptoms^c^	0.65	0.10	Beta	Bartsch et al. (2021)^19^
Hospitalized, non-pneumonia symptoms^d^	0.51	0.09	Beta	Bartsch et al. (2021)^19^
ARDS^e^	0.10	0.08–0.15	Normal	Wu et al. (2018)^24^
Disease duration				
Symptomatic days	9.00	8.61–9.39	Normal	Menni et al. (2022)^25^
Isolation days	5.00	4.61–5.39	Normal	Du et al. (2022)^26^
Hospitalization days, vaccinated	4.30	4.20–4.40	Normal	Havers et al. (2022)^27^
Hospitalization days, unvaccinated	4.60	4.50–4.70	Normal	Havers et al. (2022)^27^
Death days	300.00	241.20–358.80	Normal	Assumed
Transition probabilities (monthly)				
Vax-Suscep-NotHosp to Vax-Suscep-NotHosp	0.9843	0.9833–0.9851	—	Assumed
Vax-Suscep-NotHosp to Vax-Inf-Hosp	0.0003	0.0003–0.0004	Beta	King County (2023)^28^
Vax-Suscep-NotHosp to Vax-Inf-NotHosp	0.0154	0.0146–0.0163	Beta	King County (2023)^28^
Vax-PostInf-Hosp to Vax-Suscep-NotHosp	0.9217	0.9021–0.9413	Normal	Assumed
Vax-PostInf-Hosp to Death	0.0783	0.0587–0.0979	Normal	Danza et al. (2022)^29^
Unvax-Suscep-NotHosp to Unvax-Suscep-NotHosp	0.9565	0.9533–0.9589	—	Assumed
Unvax-Suscep-NotHosp to Unvax-Inf-NotHosp	0.0370	0.0352–0.0393	Beta	King County (2023)^28^
Unvax-Suscep-NotHosp to Unvax-Inf-Hosp	0.0065	0.0059–0.0075	Beta	King County (2023)^28^
Unvax-PostInf-Hosp to Unvax-Suscep-NotHosp	0.8784	0.8588–0.8980	Normal	Assumed
Unvax-PostInf-Hosp to Death	0.1216	0.1020–0.1412	Normal	Danza et al. (2022)^29^
Population parameters				
% vaccinated	0.75	0.20–0.90	Normal	Cox et al. 2022^15^
% aged 18–64 years	0.92	—	—	Rogers et al. (2023)^14^
% aged ≥65 years	0.08	—	—	Rogers et al. (2023)^14^
Characteristics of care seeking and care				
% screened: PCR testing	0.80	0.70–0.90	Normal	Assumed
% screened: Ag testing	0.80	0.70–0.90	Normal	Assumed
% symptomatic				
Vax-Suscep-NotHosp	0.15	0.11–0.19	Normal	Rogers et al. (2023)^14^
Vax-Inf-Hosp	1.00	0.95–1.00	—	Assumed
Vax-Inf-NotHosp	0.60	0.52–0.68	Normal	Cox et al. (2023)^30^
Vax-PostInf-Hosp	1.00	0.95–1.00	—	Assumed
Vax-PostInf-NotHosp	0.30	0.24–0.36	Normal	Cox et al. (2023)^30^
Unvax-Suscep-NotHosp	0.15	0.11–0.19	Normal	Rogers et al. (2023)^14^
Unvax-Inf-NotHosp	0.70	0.62–0.78	Normal	Cox et al. (2023)^30^
Unvax-Inf-Hosp	1.00	0.95–1.00	—	Assumed
Unvax-PostInf-NotHosp	0.35	0.29–0.41	Normal	Cox et al. (2023)^30^
Unvax-PostInf-Hosp	1.00	0.95–1.00	—	Assumed
% seeking outpatient care if symptomatic	0.33	0.29–0.37		Cox et al. (2023)^30^
% in ICU				
Vaccinated	0.20	0.18–0.21	Normal	Havers et al. (2022)^27^
Unvaccinated	0.22	0.20–0.24	Normal	Havers et al. (2022)^27^
Test performance				
PCR sensitivity asymptomatic	0.80	0.78–0.82	Normal	Srivatsan et al. (2022)^31^
PCR sensitivity symptomatic	0.90	0.88–0.92	Normal	Kortela et al. (2021)^32^
PCR specificity asymptomatic	1.00	0.99–1.00	Beta	Skittrall et al. (2021)^33^
PCR specificity symptomatic	0.99	0.98–1.00	Beta	Srivatsan et al. (2022)^31^
Ag sensitivity asymptomatic	0.28	0.21–0.34	Normal	Venekamp et al. (2023)^34^
Ag sensitivity symptomatic	0.79	0.75–0.83	Normal	Schuit et al. (2022)^35^
Ag specificity asymptomatic	1.00	0.99–1.00	Beta	Venekamp et al. (2023)^34^
Ag specificity symptomatic	0.97	0.92–1.00	Beta	Schuit et al. (2022)^35^

*Note:* Although the study population is unique, and utility may deviate from the general population, these sources most closely represent standard utility for adults in the U.S.

a Mild COVID-19 symptoms; assumes 200 mg of ibuprofen or acetaminophen orally every 4–6 hours as needed.

b Moderate COVID-19 symptoms.

c Uses influenza without hospitalization as a proxy.

d Uses influenza with hospitalization as a proxy.

e ARDS assumes required ventilator use in ICU. Ag, antigen; ARDS, acute respiratory distress syndrome; CMS, Centers for Medicare & Medicaid Services’; ICU, intensive care unit; PCR, polymerase chain reaction; QALY, quality-adjusted life year; SFS, Seattle Flu Study; USD, U.S. dollars.

An intervention was considered to be cost-effective if the incremental cost-effectiveness ratio (ICER) was ≤$150,000 per quality-adjusted life year (QALY) and economically dominant if it saved costs and provided health effects (i.e., cost saving).^37^, ^38^, ^39^ The 1-year time horizon did not account for premature mortality or lifetime productivity losses. In addition, it was assumed that COVID-19 health impacts did not extend past 1 year. Children aged <18 years and shelter staff were excluded in this analysis. Voluntary participation in surveillance interventions was assumed. Results are presented from both a healthcare payer and limited societal perspectives.

### Study Population, Setting, and Location

This study modeled homeless shelter residents in King County, Washington. Population and symptom characteristics were parameterized using input data from shelter residents within the SFS population aged ≥18 years (Table 1^14^^,^^15^^,^^18^, ^19^, ^20^, ^21^, ^22^, ^23^, ^24^, ^25^, ^26^, ^27^, ^28^, ^29^, ^30^, ^31^, ^32^, ^33^, ^34^, ^35^ and Appendix Figure 2 and Appendix Table 1, available online). Details about the SFS methods are included in the Appendix (available online) and published elsewhere.^11^^,^^12^^,^^14^^,^^15^^,^^40^ In brief, the study included residents from 23 shelters, including adult mixed-gender, adult male-only, adult female-only, family, and young adult (i.e., residents aged 18–25 years) shelters, selected to be sociodemographically representative of people experiencing sheltered homelessness in Seattle King County. The median age of all shelter residents was 37 years (80% adults, 20% children aged <18 years); most self-identified as male (64%), 46% experienced chronic homelessness (duration ≥1 year), and 18% were employed.^14^ Ethics approval for the SFS was obtained from the University of Washington Human Subjects Division (STUDY00007800). Because this analysis used only aggregate, published data, no additional IRB review was required.

### Costs

Costs were estimated using standardized sources, literature review, and expert opinion (Table 1^14^^,^^15^^,^^18^, ^19^, ^20^, ^21^, ^22^, ^23^, ^24^, ^25^, ^26^, ^27^, ^28^, ^29^, ^30^, ^31^, ^32^, ^33^, ^34^, ^35^). For example, over-the-counter medication costs were estimated through Walgreens Price Listing,^41^ and hospitalization costs were obtained from HHS Healthcare Cost and Utilization Project.^21^ Productivity losses due to absenteeism from work for COVID-19 illness or death were estimated using the median annual national occupational wage from the Bureau of Labor Statistics. Market wages, as opposed to nonmarket wages, were used for people of all ages to expressly value the time of all shelter residents (regardless of employment status) in order not to be discriminatory.^42^ Both net costs and incremental net costs account for the additional expenses associated with each testing intervention (e.g., PCR/Ag tests) as well as the costs that are avoided (e.g., medical expenses, productivity losses). All costs were reported in 2023 U.S. dollars, converting all costs using the Consumer Price Index inflator.^43^

The healthcare payer perspective included direct medical costs (e.g., testing, outpatient care, hospitalization), whereas the limited societal perspective additionally included both direct and indirect costs outside the healthcare sector (e.g., productivity losses related to absenteeism, out-of-pocket costs for over-the-counter medication). Intervention start-up costs (e.g., research and development of intervention materials) were excluded so that all interventions were evaluated and compared as if operating under steady-state conditions. Given the short 1-year time horizon, neither health outcomes nor costs were discounted.

### Outcomes

Health outcomes were measured in QALYs, which encompass both morbidity and mortality (i.e., the length and quality of life) into a single measure of health benefit. Healthy utility weights (representing a value 0–1 on the spectrum between death and perfect health)^44^ were lower for the proportion of residents aged ≥65 years and with underlying medical conditions. COVID-19 cases lost QALYs on the basis of age-dependent healthy QALY values and infection-specific utility weights for the number of days in each state, including symptomatic (versus asymptomatic) illness, hospitalization (versus no hospitalization), and death (versus live). Although this study population has distinctive characteristics (e.g., underlying medical conditions) and utility may deviate from the general population, standardized sources that most closely represent standard utility for U.S. adults were used for model inputs.^19^^,^^23^^,^^24^

### Sensitivity Analyses

For each scenario and perspective, 1-way sensitivity analyses were conducted. In addition, for each test type and perspective, both lower-bound and upper-bound scenario analyses were evaluated that used the 10 most influential parameters leading to a decreased (i.e., lower-bound, optimistic) ICER or an increased (i.e., upper-bound, pessimistic) ICER, respectively (Appendix Table 2, available online). For example, the upper-bound pessimistic scenario analysis for Ag testing from the payer perspective used high vaccination coverage, high test specificity, and minimal difference in utility between those who were symptomatic and those who were asymptomatic.

Finally, probabilistic sensitivity analyses were conducted by varying all inputs across their range in 5,000 simulations to generate 95% credible range. Cost-effectiveness acceptability curves were plotted to show the probability of each testing intervention being cost-effective at different willingness-to-pay thresholds and under different perspectives.

### Engagement Approach

Partnerships with local shelter management and public health leaders were established prior to the COVID-19 pandemic, allowing for community engagement and input throughout the research process. During SFS data collection, the authors had regular check-ins with shelter staff, allowing for the incorporation of resident and staff feedback and quick dissemination of findings to inform real-time decision making locally. Findings from this analysis will be shared through community networks with the goal of creating a more effective and equitable response to the COVID-19 pandemic among people experiencing homelessness. Furthermore, the evidence gathered through this analysis may be translated to plan for future pandemic preparedness and resource allocation.

## RESULTS

Table 1^14^^,^^15^^,^^18^, ^19^, ^20^, ^21^, ^22^, ^23^, ^24^, ^25^, ^26^, ^27^, ^28^, ^29^, ^30^, ^31^, ^32^, ^33^, ^34^, ^35^ displays key model inputs, including costs, QALY estimates, and transition probabilities. Where possible, the authors used primary data from the SFS specific to homeless shelter residents in King County. Data were supplemented from published studies on COVID-19 and previous models evaluating COVID-19 testing strategies in the U.S. (Appendix Table 3, available online).

The estimated costs of PCR and Ag tests were $19.56 (range=$17.60–$21.52) and $5.50 (range=$4.52–$6.48), respectively. Operational costs of PCR testing ($18.66; range=$16.70–$20.62) were also greater than that of Ag testing ($4.98; range=$4.00–$5.96) owing to the need to transport and process specimens and return results.^18^^,^^45^^,^^46^ Utility weights for healthy QALY estimates were age specific (0.92 for those aged 18–64 years; 0.84 for those aged ≥65 years).^23^ Influenza without hospitalization was used as a proxy for the utility weight of mild nonspecific COVID-19 symptoms (0.648),^47^, ^48^, ^49^, ^50^, ^51^ whereas influenza with hospitalization was used as a proxy for utility weight of hospitalized, non-pneumonia COVID-19 (0.514).^49^^,^^51^^,^^52^

All transition probabilities were estimated from data during the Omicron phase of the COVID-19 pandemic in the U.S. from November 27, 2021, to March 27, 2022, stratified by COVID-19 vaccination status.^28^^,^^29^ Transition probabilities from susceptible to infected states were King County specific and adjusted for the proportion and 95% uncertainty interval of cases or hospitalizations reported.^28^^,^^53^ The proportion estimated to be symptomatic in each state was derived from previously published SFS data on self-reported symptoms among shelter residents.^14^^,^^30^

Table 2 summarizes the results comparing Ag testing with no surveillance and PCR with Ag testing from both the healthcare payer and societal perspectives. The majority of rapid Ag testing scenarios were cost-effective, whereas PCR testing was either not cost-effective or dominated by (i.e., less effective and more costly than) Ag testing.

**Table 2:** Effectiveness and Cost-Effectiveness of Expanded COVID-19 Surveillance Strategies by Vaccination Coverage: Ag Testing Versus No Surveillance and PCR Testing Versus Ag Testing

Testing scenario	Incremental net cost (2023 USD)	Incremental effectiveness (QALYs gained)	ICER ($/QALY gained)
Healthcare payer perspective			
1. No surveillance	ref	ref	ref
2. Ag testing^a^			
**75% vaccination coverage**	**$97.42**	**0.00087**	**$112,350.52**
20% vaccination coverage	$80.39	0.00266	$30,196.45
90% vaccination coverage	$100.94	0.00053	$189,912.51
95% credible range^c^	*($76.72, $119.24)*	*(0.00012, 0.00228)*	*($37,149.09, $817,144.09)*
3. PCR testing^b^			
**75% vaccination coverage**	**$288.14**	**0.00002**	**$12,852,158.53**
20% vaccination coverage	$286.22	0.00026	$1,120,147.96
90% vaccination coverage	$288.39	−0.000003	Dominated
95% credible range^c^	*($228.59, $347.83)*	*(−0.00045, 0.00050)*	*(Dominated, $37,875,912.35)*
Limited societal perspective			
1. No surveillance	ref	ref	ref
2. Ag testing^a^			
**75% vaccination coverage**	**$8.35**	**0.00087**	**$9,626.79**
20% vaccination coverage	−$103.67	0.00266	Dominant
90% vaccination coverage	$36.98	0.00053	$69,574.33
95% credible range^c^	*(−$170.54, $128.85)*	*(0.00012, 0.00228)*	*(Dominant, $520,424.88)*
3. PCR testing^b^			
**75% vaccination coverage**	**$294.26**	**0.00002**	**$13,125,040.73**
20% vaccination coverage	$313.98	0.00026	$1,228,785.26
90% vaccination coverage	$288.35	−0.000003	Dominated
95% credible range^c^	*($164.98, $420.00)*	*(−0.00045, 0.00050)*	*(Dominated, $38,513,686.83)*

*Note:* Baseline scenario of 75% vaccination coverage bolded.

a Ag testing compared with no surveillance.

b PCR testing compared with Ag testing.

c 95% credible range was calculated by computing the 2.5th and 97.5th percentiles across the 5,000-model simulation runs in probabilistic sensitivity analyses. Ag, antigen; ICER, incremental cost-effectiveness ratio; PCR, polymerase chain reaction; QALY, quality-adjusted life year; USD, U.S. dollars.

**Figure 2:**
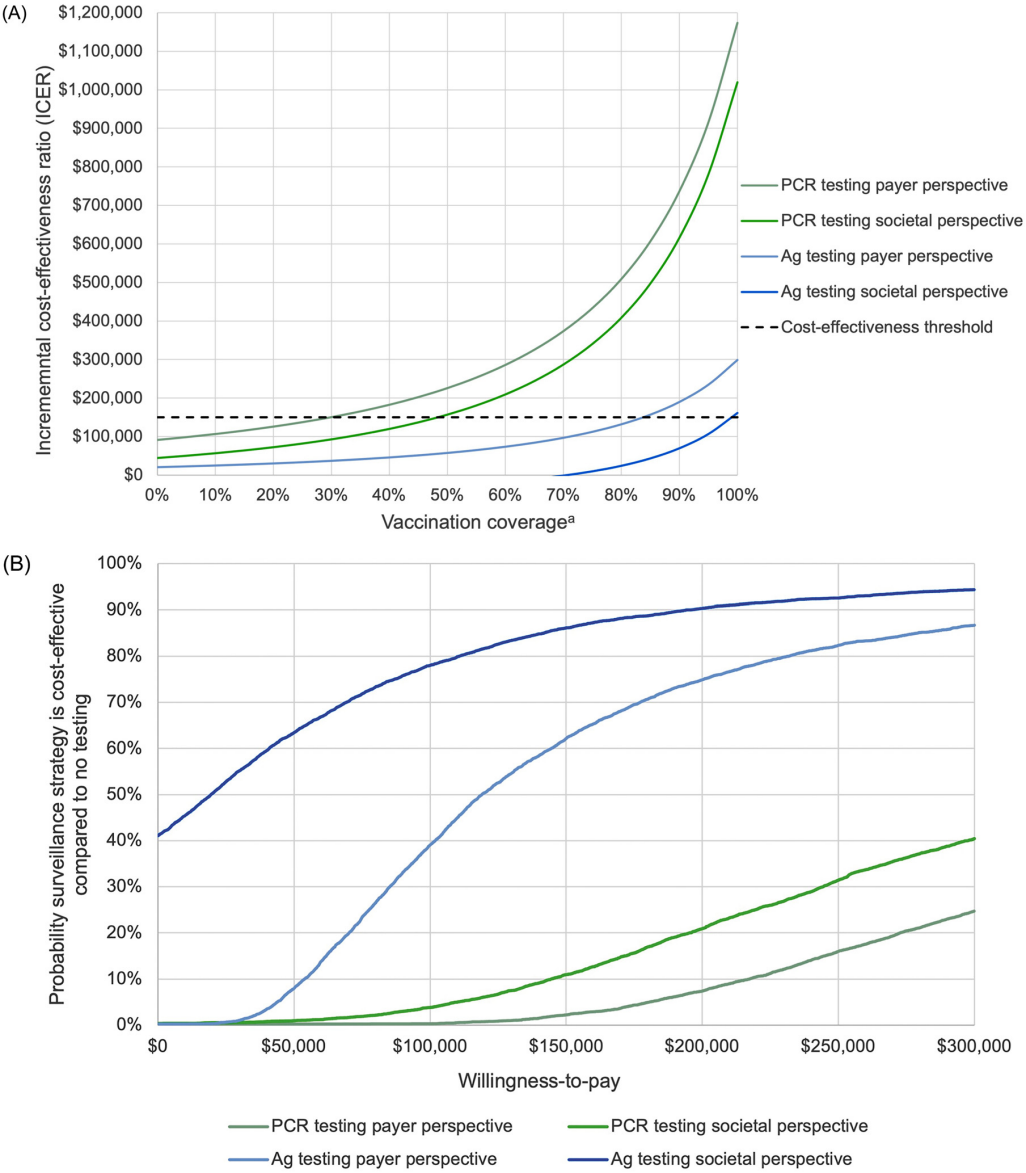
Cost-effectiveness scenarios. (A) Cost-effectiveness by vaccination coverage, test type, and perspective: Ag testing versus no surveillance testing and PCR testing versus no surveillance testing. All monthly COVID-19 testing strategies pictured are compared with no surveillance (PCR testing compared with Ag testing was never cost-effective). ^a^Vaccination coverage represents proportion of the population with at least 1 dose of COVID-19 vaccine. (B) Cost-effectiveness acceptability curve by COVID-19 surveillance strategy: Ag testing versus no surveillance and PCR testing versus no surveillance. All COVID-19 testing strategies pictured are compared with no surveillance (PCR testing compared with Ag testing was never cost-effective). Ag, antigen; PCR, polymerase chain reaction.

In scenarios where both Ag and PCR COVID-19 surveillance testing strategies were available (Table 2), Ag testing was cost-effective compared with no surveillance from both the healthcare payer perspective (ICER=$112,351 per QALY gained) and the societal perspective (ICER=$9,627 per QALY gained) at 75% vaccination coverage. Ag testing averted 0.027% of infections, with mean QALYs increasing by 0.0009 (95% credible range=0.0001, 0.0023), at an incremental net cost of $97 ($76, $119) and $8 (−$171, $129) per shelter resident from the healthcare payer and societal perspectives, respectively. At ≤70% vaccination coverage, Ag testing was cost-saving from the societal perspective (Figure 2A and Appendix Figure 3A and B, available online). Compared with Ag testing, PCR testing averted an additional 0.0008% of infections and increased mean QALYs by 0.00002 (−0.00045, 0.00050) at an incremental net cost of $288 ($229, $348) per shelter resident from the healthcare payer perspective and $294 ($165, $420) per shelter resident from the societal perspective (Appendix Figure 3A and B, available online). Assuming availability of both tests, PCR testing was not cost-effective from either perspective compared with Ag testing (ICER=$12,852,159 per QALY gained from the payer perspective, $13,125,041 per QALY gained from the societal perspective at 75% vaccination coverage).

In a scenario when Ag testing was not available (i.e., comparing PCR testing with no in-shelter surveillance) (Table 3), PCR testing averted 0.028% infections and increased mean QALYs by 0.0009 (95% credible range=0.0001, 0.0024) at 75% vaccination coverage, with an incremental net cost of $386 ($327, $444) per shelter resident from the healthcare payer perspective and $303 ($144, $422) per shelter resident from the societal perspective. PCR testing was not cost-effective from either perspective compared with no surveillance at 75% vaccination coverage (payer perspective: ICER of $433,441 per QALY gained; societal perspective: ICER of $340,184 per QALY gained) (Table 3). However, PCR testing was cost-effective compared with no surveillance when vaccination coverage was <30% from the healthcare perspective and ≤48% from the societal perspective (Figure 2A and Appendix Figure 3B and D, available online).

**Table 3:** Effectiveness and Cost-Effectiveness of Expanded COVID-19 Surveillance Strategies by Vaccination Coverage if No Ag Testing Available: PCR Testing Versus No Surveillance

Testing scenario	Incremental net cost (2023 USD)	Incremental effectiveness (QALYs gained)	ICER ($/QALY gained)
Healthcare payer perspective			
1. No surveillance	ref	ref	ref
2. PCR testing^a^			
**75% vaccination coverage**	**$386**	**0.00089**	**$433,441**
20% vaccination coverage	$367	0.00292	$125,651
90% vaccination coverage	$389	0.00053	$737,038
95% credible range^b^	*($327, $444)*	*(0.00012, 0.00244)*	*($153,046, $3,195,660)*
Limited societal perspective			
1. No surveillance	ref	ref	ref
2. PCR testing^a^			
**75% vaccination coverage**	**$303**	**0.00089**	**$340,183.94**
20% vaccination coverage	$210	0.00292	$72,081.42
90% vaccination coverage	$325	0.00053	$615,878.90
95% credible range^b^	*($144, $422)*	*(0.00012, 0.00244)*	*($84,331, $2,767,971)*

*Note:* Baseline scenario of 75% vaccination coverage bolded.

a PCR testing compared with no surveillance.

b 95% credible range was calculated by computing the 2.5th and 97.5th percentiles across the 5,000-model simulation runs in probabilistic sensitivity analyses. Ag, antigen; ICER, incremental cost-effectiveness ratio; PCR, polymerase chain reaction; QALY, quality-adjusted life year; USD, U.S. dollars.

Figure 2B illustrates results from probabilistic sensitivity analyses for each COVID-19 testing strategy compared with no surveillance. PCR testing was cost-effective in 2% and 11% of simulations from the payer and societal perspectives, respectively. However, Ag testing was cost-effective in 62% and 86% of simulations from the payer and societal perspectives, respectively.

Tornado diagrams of one-way sensitivity analyses for PCR testing compared with no surveillance and Ag testing compared with no surveillance are presented in Appendix Figure 4A–D (available online). In both scenarios from the healthcare payer perspective, the ICER was most sensitive to uncertainty in the proportion vaccinated, utility weights, test specificity, and test cost (Appendix Figure 4A and B, available online). From the societal perspective, uncertainty in the daily wage, proportion screened, and proportion symptomatic among infected and nonhospitalized individuals had a large impact on the ICER (Appendix Figure 4C and D, available online). When varying the proportion of residents screened for COVID-19 between 0% and 100%, Ag testing (versus no surveillance) was always cost-effective or cost-saving, whereas PCR testing (versus no surveillance) became cost-effective at a threshold of ≤2% and ≤50% screening from the payer and societal perspectives, respectively (Appendix Figure 5, available online).

No scenario analyses of PCR testing compared with Ag testing resulted in cost-effective ICERs (Appendix Table 4, available online). When compared with no surveillance, upper-bound, pessimistic scenario analyses showed that ICERs for all test types and perspectives have potential to surpass the $150,000 cost-effectiveness threshold. However, for both PCR and Ag tests, lower-bound optimistic scenario analyses illustrated that the ICERs from the payer perspective can be cost-effective, whereas ICERs from the societal perspective can be cost-saving (Appendix Tables 4 and 5, available online).

## DISCUSSION

This study estimated the health and economic impact of pandemic COVID-19 surveillance testing across King County shelters and found that most surveillance scenarios using rapid Ag testing were cost-effective from both the healthcare payer and societal perspectives. However, PCR testing was only cost-effective in the absence of Ag testing at lower vaccination coverage levels. Across scenarios, the overall cost per resident was lower from the societal perspective than from the healthcare payer perspective owing to the inclusion of productivity losses, which offset testing costs. Study results were most sensitive to parameters such as the proportion of the population vaccinated, utility weights, test specificity, and daily wage. Obtaining precise and timely estimates for these parameters is crucial to limiting uncertainty in future research. These modeled findings can inform public health agency decisions and have policy implications regarding the costs and benefits of COVID-19 pandemic surveillance to society.

Previous modeling studies of COVID-19 surveillance were conducted before the availability of COVID-19 vaccines and mostly found that surveillance was cost-effective or cost-saving (Appendix Table 3, available online).^26^^,^^45^^,^^54^, ^55^, ^56^, ^57^, ^58^, ^59^, ^60^, ^61^ For example, Maya et al.^55^ found that Ag testing had the potential to be dominant (cheaper and more effective) over PCR-only testing in a population of healthcare workers in the U.S. Appendix Table 3 (available online) summarizes published, peer-reviewed cost-effectiveness analyses evaluating various COVID-19 testing strategies in U.S. settings identified using a systematic review by Zhou and colleagues (2022),^62^ supplemented with an updated search of articles published since July 2023. Of the 10 analyses, 3 used a decision tree model,^45^^,^^54^^,^^55^ 5 used a compartmental model,^56^, ^57^, ^58^, ^59^, ^60^ and 2 used a stochastic agent–based model.^26^^,^^61^ The median time horizon reported was 136 days, ranging from 60 to 270 days. The majority of analyses used ICERs to evaluate cost-effectiveness. This study utilized a Markov model, enabling evaluation of repeat testing and repeat infection as well as dynamic incidence to account for reduced transmission due to isolation.

This study adds to the minimal data available on the value of in-shelter PCR and Ag surveillance for COVID-19 among adults experiencing homelessness and the impact of different test types. These findings can be used by shelter management and public health jurisdictions to inform the implementation of COVID-19 surveillance testing. The results may also be relevant to other shelter settings or similar congregate living settings (e.g., prisons or border detention centers) in current or future seasonal epidemics.^63^, ^64^, ^65^ In addition, this model could serve as a foundation for simulating other respiratory virus outbreak scenarios by updating model parameters.

### Limitations

This analysis had several limitations. First, the model did not include waning immunity of vaccination over time; instead, a combination of vaccination coverage and infection transition probabilities was used and varied in sensitivity analyses. However, even under the conservative scenario of no immunity from vaccination (i.e., 0% vaccination coverage), all testing scenarios were cost-effective or cost-saving compared with no surveillance testing. Second, children were excluded from the analysis because of limited available data on vaccination coverage and COVID-19 transmission dynamics among children in shelters. Future research should incorporate subgroup analysis to understand differences between key subpopulations (e.g., children versus adults, staff versus residents, types of shelters) for a more comprehensive assessment of the overall impact of testing interventions. In addition, although the model aimed to incorporate the most recent and applicable model parameters (e.g., regional statistics since the Omicron wave of the pandemic), the analysis was limited by available data. Ongoing monitoring of COVID-19 is necessary to incorporate the most relevant and accurate data to ensure that findings remain actionable for public health decision making in the present and future contexts.

## CONCLUSIONS

This is a novel cost–utility analysis comparing PCR with Ag COVID-19 testing strategies among people experiencing sheltered homelessness during the COVID-19 pandemic. Estimates of cost-effectiveness are conservative as the Markov model excludes herd immunity and only crudely incorporates transmission dynamics by adjusting transition probabilities on the basis of the proportion of individuals isolated. Future models should consider incorporating transmission using a dynamic model to better capture these health benefits. In addition, the 1-year time horizon was considered to be conservative because it did not account for premature mortality or lifetime productivity losses due to COVID-19; over a longer time horizon, screening would avert COVID-19 cases and long COVID, likely leading to decreased costs and increased utility. Capturing long-term benefits of avoiding COVID-19 and accounting for changes in viral variants (e.g., transmissibility), test availability, and population behavior (e.g., vaccination) will be crucial for future analyses to ensure the relevance and applicability of findings. As the COVID-19 landscape evolves, this analysis should be revisited with updated parameters, particularly those with greatest influence where there is large uncertainty such as the proportion vaccinated, test cost, and test performance. Model updates and comprehensive reporting of updated sensitivity analyses will allow decision makers to assess the validity of the model as well as guide future data collection efforts to obtain more precise parameter estimates.^66^

Rapid Ag testing for COVID-19 in congregate settings is likely to be cost-effective, whereas PCR testing may be optimal at low vaccination coverage levels if Ag testing is not available. This study can help decision makers in shelter populations and other congregate settings develop recommendations for surveillance implementation to reduce morbidity and mortality.

## Appendix Supplementary materials

Image, application 1

## Footnotes

Supplementary material associated with this article can be found in the online version at doi:10.1016/j.focus.2024.100307.
