# Supplementary material for: Health and Economic Impact of COVID-19 Surveillance Testing in Seattle Homeless Shelters: A Cost-Effectiveness Analysis
Source: AJPM Focus. 2024 Dec 5;4(2):100307. doi: 10.1016/j.focus.2024.100307 (PMC11889550; doi:10.1016/j.focus.2024.100307)
Supplement: Supplementary file 1 [file mmc1.docx]

**APPENDIX**

***Health and Economic Impact of COVID-19 Surveillance Testing in Seattle Homeless Shelters: A Cost-Effectiveness Analysis***

**Appendix** [**Table 1.** Seattle Flu Study shelter sites where sample collection occurred, 1 January 2020 – 31 May 2021 1](#_Toc182659979)

[**Appendix Table 2.** Model inputs for scenario sensitivity analyses 3](#_Toc182659980)

[**Appendix Table 3.** Published, peer-reviewed cost-effectiveness analyses evaluating various SARS-CoV-2 testing strategies in U.S. settings^1–10^ 5](#_Toc182659981)

[**Appendix Table 4.** Effectiveness and cost-effectiveness of expanded COVID-19 surveillance strategies by vaccination coverage: lower bound and upper bound scenario analyses results: Ag testing vs. no surveillance, PCR testing vs. Ag testing 7](#_Toc182659982)

[**Appendix Table 5.** Effectiveness and cost-effectiveness of expanded COVID-19 surveillance strategies by vaccination coverage: lower bound and upper bound scenario analyses results: PCR testing vs. no surveillance 8](#_Toc182659983)

[**Appendix Figure 1.** Data analysis plan 9](#_Toc182659984)

[**Appendix Figure 2.** Map of Seattle Flu Study Shelter Locations 10](#_Toc182659985)

[**Appendix Figure 3.a.** Cost per adult shelter resident by vaccine coverage, test type, and perspective: Ag testing vs. no surveillance, PCR testing vs. Ag testing 11](#_Toc182659986)

[**Appendix Figure 3.b.** Cost per adult shelter resident by vaccine coverage, test type, and perspective: Ag testing vs. no surveillance, PCR testing vs. no surveillance 12](#_Toc182659987)

[**Appendix Figure 3.c.** Quality-adjusted life years per adult shelter resident by vaccine coverage, test type, and perspective: Ag testing vs. no surveillance, PCR testing vs. Ag testing 13](#_Toc182659988)

[**Appendix Figure 3.d.** Quality-adjusted life years per adult shelter resident by vaccine coverage, test type, and perspective: Ag testing vs. no surveillance, PCR testing vs. no surveillance 14](#_Toc182659989)

[**Appendix Methods.** Description of Seattle Flu Study (SFS) 18](#_Toc182659990)

### **Appendix Table 1.** Seattle Flu Study shelter sites where sample collection occurred, 1 January 2020 – 31 May 2021

| Shelter | Maximum capacity | Resident sex | Resident age range | Sleeping arrangements available |
| --- | --- | --- | --- | --- |
| A | 60 | Female | ≥ 18 years | Communal bunk beds |
| B | 100 | Mixed | ≥ 18 years | Communal bunk beds |
| C | 45 | Mixed | 18 - 25 years | Communal floor mats and bunks beds |
| D | 185 | Mixed | All ages ^+^ | Private rooms / shared rooms / communal floor mats |
| E | 70 | Mixed | All ages ^+^ | Private rooms / shared rooms / communal floor mats |
| F | 60 | Male | ≥ 18 years | Communal bunk beds |
| G | 275 | Mixed | ≥ 18 years | Private rooms / shared rooms |
| H | 275 | Mixed | All ages ^+^ | Private rooms / shared rooms |
| I | 45 | Male | ≥ 50 years | 5 person dorms |
| J* | 34 | Male | ≥ 18 years | Individual open cubicles |
| K** | 75 | Mixed | ≥ 18 years | Individual open cubicles |
| L | 200 | Mixed | ≥ 18 years | Communal bunk beds |
| M | 212 | Male | ≥ 50 years | Communal floor mats |
| N | 46 | Mixed | All ages ^+^ | Private rooms / shared rooms |
| O | 100 | Mixed | All ages^+^ | Private rooms / shared rooms / communal floor mats |
| P | 100 | Male | ≥ 50 years | Communal floor mats |
| Q | 100 | Mixed | ≥ 18 years | Private apartments |
| R | 150 | Mixed | ≥ 18 years | Communal floor mats |
| S | 234 | Mixed | ≥ 18 years | Private apartments |
| T | 49 | Male | ≥ 50 years | Communal floor mats |
| U | 50 | Mixed | All ages^+^ | Private rooms / shared rooms / communal floor mats |
| V | 18 | Mixed | <18 years | Communal bunk beds |
| W | 20 | Mixed | 18 - 25 years | Communal bunk beds |

^+^All ages= family shelter

^*^*Opened / data collection began 3 December 2020 to replace Shelter F*

*^**^ Opened / data collection began 3 December 2020 to replace Shelter B*

### **Appendix Table 2.** Model inputs for scenario sensitivity analyses

| **Top 10 most influential parameters** | | **Lower bound input (optimistic ICER)** | **Upper bound input (pessimistic ICER)** |
| --- | --- | --- | --- |
| ***Scenario analysis 1: PCR payer*** | | ***1a lower bound*** | ***1b upper bound*** |
| 1 | Proportion vaccinated | 0.20 | 0.90 |
| 2 | Utility when symptomatic | 0.55 | 0.75 |
| 3 | Utility when asymptomatic | 0.99 | 0.84 |
| 4 | PCR Specificity Asymptomatic | 0.99 | 1.00 |
| 5 | PCR Specificity Symptomatic | 0.98 | 1.00 |
| 6 | Cost of PCR test | $17.60 | $21.52 |
| 7 | Cost of PCR test implementation | $16.70 | $20.62 |
| 8 | Proportion symptomatic among Vax-Inf-Nothosp | 0.6784 | 0.5216 |
| 9 | Monthly probability of getting COVID + being hospitalized for COVID among those who were unvaccinated | 0.00745 | 0.00586 |
| 10 | Monthly probability of getting COVID (unhospitalized) among those who were vaccinated | 0.02 | 0.01 |
| ***Scenario analysis 2: PCR societal*** | | ***2a lower bound*** | ***2b upper bound*** |
| 1 | Proportion vaccinated | 0.20 | 0.90 |
| 2 | Utility when symptomatic | 0.55 | 0.75 |
| 3 | Utility when asymptomatic | 0.99 | 0.84 |
| 4 | Cost of daily lost productivity (daily wage) | $479.76 | $118.93 |
| 5 | Proportion screened using PCR test | 0.70 | 0.90 |
| 6 | Proportion symptomatic among Vax-Inf-Nothosp | 0.68 | 0.52 |
| 7 | Proportion symptomatic among Unax-Inf-Nothosp | 0.78 | 0.62 |
| 8 | Cost PCR test | $17.60 | $21.52 |
| 9 | Cost per PCR test implementation | $16.70 | $20.62 |
| 10 | Monthly probability of getting COVID (unhospitalized) among those who were vaccinated | 0.02 | 0.01 |
| ***Scenario analysis 3: Ag payer*** | | ***3a lower bound*** | ***3b upper bound*** |
| 1 | Proportion vaccinated | 0.20 | 0.90 |
| 2 | Ag Specificity Symptomatic | 0.92 | 1.00 |
| 3 | Utility when symptomatic | 0.55 | 0.75 |
| 4 | Utility when asymptomatic | 0.99 | 0.84 |
| 5 | Ag Specificity Asymptomatic | 0.99 | 1.00 |
| 6 | Cost rapid Ag test | $4.52 | $6.48 |
| 7 | Cost per Ag test implementation | $4.00 | $5.96 |
| 8 | Proportion symptomatic among Vax-Inf-Nothosp | 0.6784 | 0.5216 |
| 9 | Proportion symptomatic among Unax-Inf-Nothosp | 0.77840 | 0.6216 |
| 10 | Monthly probability of getting COVID + being hospitalized for COVID among those who were unvaccinated | 0.00745 | 0.00586 |
| ***Scenario analysis 4: Ag societal*** | | ***4a lower bound*** | ***4b upper bound*** |
| 1 | Cost of daily lost productivity (daily wage) | $479.76 | $118.93 |
| 2 | Ag Specificity Symptomatic | 1.00 | 0.92 |
| 3 | Proportion vaccinated | 0.20 | 0.90 |
| 4 | Proportion screened using Ag test | 0.70 | 0.90 |
| 5 | Ag Specificity Asymptomatic | 1.00 | 0.99 |
| 6 | Proportion symptomatic among Vax-Inf-Nothosp | 0.68 | 0.52 |
| 7 | Isolation days | 4.61 | 5.39 |
| 8 | Proportion symptomatic among Unax-Inf-Nothosp | 0.78 | 0.62 |
| 9 | Symptomatic days | 9.39 | 8.61 |
| 10 | Cost of Ag test | $4.52 | 6.48 |

### **Appendix Table 3.** Published, peer-reviewed cost-effectiveness analyses evaluating various SARS-CoV-2 testing strategies in U.S. settings^1–10^

| **Author** | **Type** | **Population** | **Perspective** | **Intervention** | **Comparator** | **Sensitivity analysis** | **Time horizons** | **Discount rate** | **Parameter index** | **Willingness to pay** | **Conclusion** |
| --- | --- | --- | --- | --- | --- | --- | --- | --- | --- | --- | --- |
| *Baggett et al. (2020)* | Compartmental model (CEACOV) | Adult homeless shelter residents | Health care system | Daily symptom screening, universal PCR testing every 2 weeks, hospital-based or alternative care sites based COVID-19 care, and temporary housing | No intervention | Yes (DSA) | 4 months (April-August 2020) | *Not reported* | ICER | $1,000/ case prevented | Daily symptom screening and alternative care sites was cost-effective (adding universal PCR testing every 2 weeks in surging epidemic) |
| *Neilan et al. (2020)* | Compartmental model (CEACOV) | General population | Health care system | PCR testing: hospital-based, symptom-based, symptom-based + asymptomatic 1x, symptom-based + asymptomatic 1x/monthly | Symptom-based testing | Yes | 180 days | 3% | ICER | $100,000/ QALY | Cost-effective |
| *Losina et al. (2021)* | Compartmental model (CEACOV) | Undergraduate students and faculty at colleges | Societal (modified) | COVID-19 mitigation strategies (social distancing, masks, & routine laboratory screening) | No intervention | Yes | 105 days  (1 semester) | *Not reported* | ICER | $100,000/ QALY | Extensive social distancing with a mandatory mask wearing policy was cost-effective, routine laboratory testing was not |
| *Paltiel et al. (2020)* | Compartmental model | Students in residential college setting | *Not reported* | Rapid testing with daily - weekly frequency (i.e., every 1, 2, 3, 7 days) | Symptom-based testing | Yes | 80 days (abbreviated semester) | *Not reported* | ICER | $5,500 ~ 11,600 /infection averted | Screening every 2 days using a rapid, inexpensive, and even poorly sensitive (>70%) test, coupled with strict behavioral interventions to keep Rt less than 2.5 was cost-effective |
| *Paltiel et al. (2021)* | Compartmental model | General population | Societal | Home-based rapid Ag testing: weekly | No intervention | Yes | 60 days | *Not reported* | ICER | $5,000,000 ~17,000,000 /VSL | Cost-effective |
| *Du et al. (2021)* | Stochastic agent-based model | General population | Societal | 8 rapid Ag testing strategies: daily - monthly frequency (i.e., every 1, 7, 14, 28 days) & isolation (1 vs. 2 weeks) | Symptom-based testing | Yes | 150 days | A discrete-time discount factor | NMB | $100,000/ YLL averted | Cost-effective |
| *Du et al (2022)* | Stochastic agent-based model | Population in a typical US community | Societal | 6,651 rapid mass proactive Ag testing strategies: daily - monthly frequency (i.e., every 1, 7, 28 days) & isolation | No intervention (alt: status quo baseline symptomatic testing) | Yes | 150 days | *Not reported* | NMB | $100,000/ averted YLL | Cost-effective |
| *Savitsky et al. (2020)* | Decision tree model | Health care workers | *Not reported* | Universal rapid testing | Universal PPE use | Yes (DSA and PSA) | *Not reported* | None | ICER | $25,000 /infection averted | Universal COVID-19 screening is generally the preferred option. However, universal PPE may be cost-effective and preferred in locations with high COVID-19 prevalence |
| *Maya et al. (2022)* | Decision tree model | Health care workers | *Not reported* | (1) only PCR test, (2) only Ag test (3) only IgG test, (4) conditional PCR test if IgG test is positive, and (5) concurrent IgG and PCR tests. | No tests | Yes (DSA and PSA) | *Not reported* | 3% | ICER | - | Both PCR and Ag testing are beneficial strategies |
| *Maya et al. (2022)* | Decision tree model | Students, teachers, staff in K-12 Schools | Societal; School administration | (1) 1x/week Ag test (2) 2x/week Ag test (3) 1x/week PCR test (4) 1x/week Ag test + confirmatory PCR  (5) 2x/week Ag test + confirmatory PCR | No tests | Yes (DSA and PSA) | 9 months (April–December 2021) | 3% | ICER | - | Cost-effective except Ag test(s) alone |

### **Appendix Table 4.** Effectiveness and cost-effectiveness of expanded COVID-19 surveillance strategies by vaccination coverage: lower bound and upper bound scenario analyses results: Ag testing vs. no surveillance, PCR testing vs. Ag testing

|  |  |  | | |
| --- | --- | --- | --- | --- |
| **Testing scenario** | | **Incremental Cost (2023 USD)** | **Incremental Effectiveness  (QALYs gained)** | **ICER  ($/QALY gained)** |
| ***Healthcare payer perspective*** | |  |  |  |
| 1. No surveillance | | Ref | Ref | Ref |
| 2. Ag testing* | Scenario analysis 3a (Ag payer lower bound) | $47 | 0.00655 | $7,211 |
|  | Scenario analysis 3b (Ag payer upper bound) | $123 | 0.00012 | $1,022,675 |
| 3. PCR testing^ | Scenario analysis 1a, 3a (PCR payer lower bound) | $320 | -0.00060 | Dominated |
|  | Scenario analysis 1b, 3b (PCR payer upper bound) | $366 | 0.00005 | $7,731,342.73 |
| ***Limited societal perspective*** | |  |  |  |
| 1. No surveillance | | Ref | Ref | Ref |
| 2. Ag testing* | Scenario analysis 4a (Ag societal) lower bound | -$723 | 0.00201 | Dominant |
|  | Scenario analysis 4b (Ag societal) upper bound | $216 | 0.00094 | $229,246.52 |
| 3. PCR testing^ | Scenario analysis 2a, 4a (PCR societal) lower bound | $375 | 0.00107 | $350,601.91 |
|  | Scenario analysis 2b, 4b (PCR societal) upper bound | $247 | -0.00022 | Dominated |

* Ag testing compared to no surveillance

^ PCR testing compared to Ag testing

### **Appendix Table 5.** Effectiveness and cost-effectiveness of expanded COVID-19 surveillance strategies by vaccination coverage: lower bound and upper bound scenario analyses results: PCR testing vs. no surveillance

|  |  |  | | |
| --- | --- | --- | --- | --- |
| **Testing scenario** | | **Incremental Cost (2023 USD)** | **Incremental Effectiveness  (QALYs gained)** | **ICER  ($/QALY gained)** |
| ***Healthcare payer perspective*** | |  |  |  |
| 1. No surveillance | | Ref | Ref | Ref |
| 2. PCR testing* | Scenario analysis 1a (PCR payer lower bound) | $323 | 0.00461 | $70,019 |
|  | Scenario analysis 1b (PCR payer upper bound) | $431 | 0.00020 | $2,173,701 |
| ***Limited societal perspective*** | |  |  |  |
| 1. No surveillance | | Ref | Ref | Ref |
| 2. PCR testing* | Scenario analysis 2a (PCR societal lower bound) | -$301 | 0.00401 | Dominant |
|  | Scenario analysis 2b (PCR societal upper bound) | $477 | 0.00023 | $2,099,511 |

* PCR testing compared to no surveillance

### **Appendix Figure 1.** Data analysis plan

| We conducted a cost-utility analysis comparing a baseline surveillance scenario consisting of a no in-shelter surveillance strategy to two alternative strategies where shelter management actively encouraged all residents & staff to participate in testing with:  1) polymerase chain reaction (PCR) testing, or 2) rapid antigen (Ag) testing. | |
| --- | --- |
| *Preliminary analysis plan (complete)* | *Secondary analysis (complete)* |
| In preliminary analyses, we developed a decision tree model to conduct the cost-utility analysis from both the healthcare payer and limited societal perspective over a one-year time horizon. Model inputs utilized data from congregate shelter residents aged ≥18 years who participated in the SFS between January 1, 2020 – May 31, 2021. The primary health outcome was quality-adjusted life-years (QALYs) gained due to averting COVID-19 disease. Healthcare and societal costs were estimated from standardized sources and literature review. All costs were converted to 2022 US dollars. We considered an intervention to be cost-effective if the incremental cost-effectiveness ratio (ICER) is $150,000/QALY and dominant if it saves costs and provides health effects. We addressed uncertainty with deterministic one-way sensitivity analyses. | In secondary analyses, we built a Markov model that incorporates dynamic incidence to simulate the transmission of SARS-CoV-2 in the congregate shelter population, to evaluate cost-utility over a one-year time horizon. Model inputs continued to utilize data from shelter residents aged ≥18 years who participated in the Seattle Flu Study (January 1, 2020 – May 31, 2021) and the primary health outcome will be quality-adjusted life-years (QALYs) gained due to averting COVID-19 disease. Healthcare and societal costs were estimated from standardized sources, literature review, and expert opinion. All costs were converted to 2023 US dollars. We considered an intervention to be cost-effective if the incremental cost-effectiveness ratio (ICER) is $150,000/QALY and dominant if it saves costs and provides health effects. We evaluated scenarios at various vaccination levels ranging from 20%-90%. We addressed uncertainty using both deterministic one-way sensitivity analyses and probabilistic sensitivity analyses. |

### **Appendix Figure 2.** Map of Seattle Flu Study Shelter Locations

| **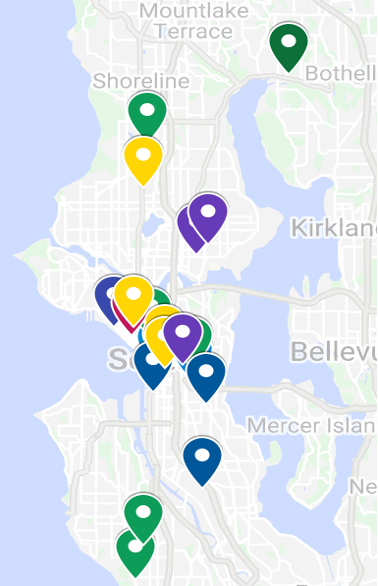** | \| **Legend:**  **Adult mixed gender shelters**  **Adult male shelters**  **Adult female shelters**  **Family shelters**  **Young adult shelters** \| \| --- \| |
| --- | --- | --- |

### **Appendix Figure 3.a.** Cost per adult shelter resident by vaccine coverage, test type, and perspective: Ag testing vs. no surveillance, PCR testing vs. Ag testing


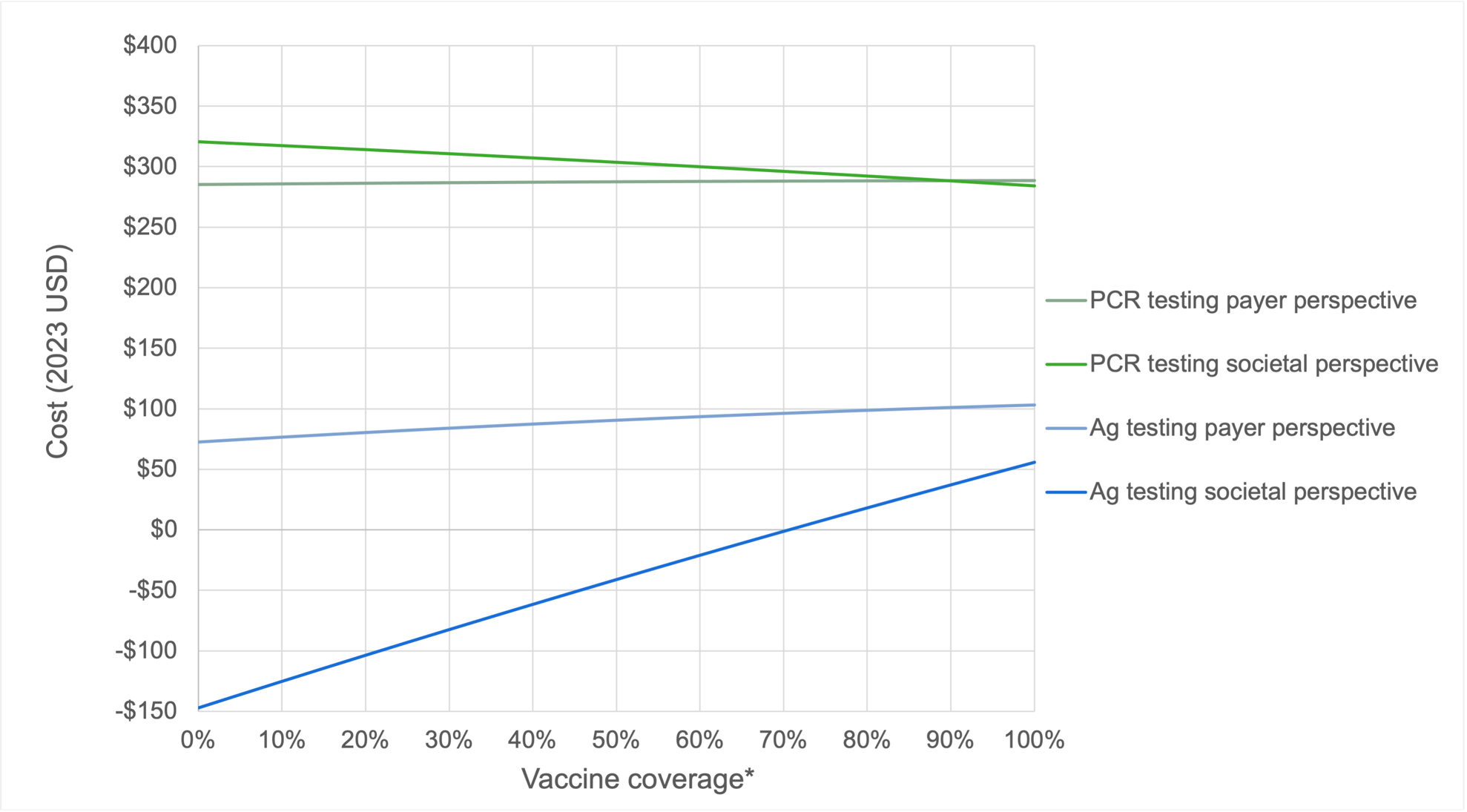
*Vaccine coverage represents proportion of the population with at least one dose of COVID-19 vaccine

### **Appendix Figure 3.b.** Cost per adult shelter resident by vaccine coverage, test type, and perspective: Ag testing vs. no surveillance, PCR testing vs. no surveillance

**
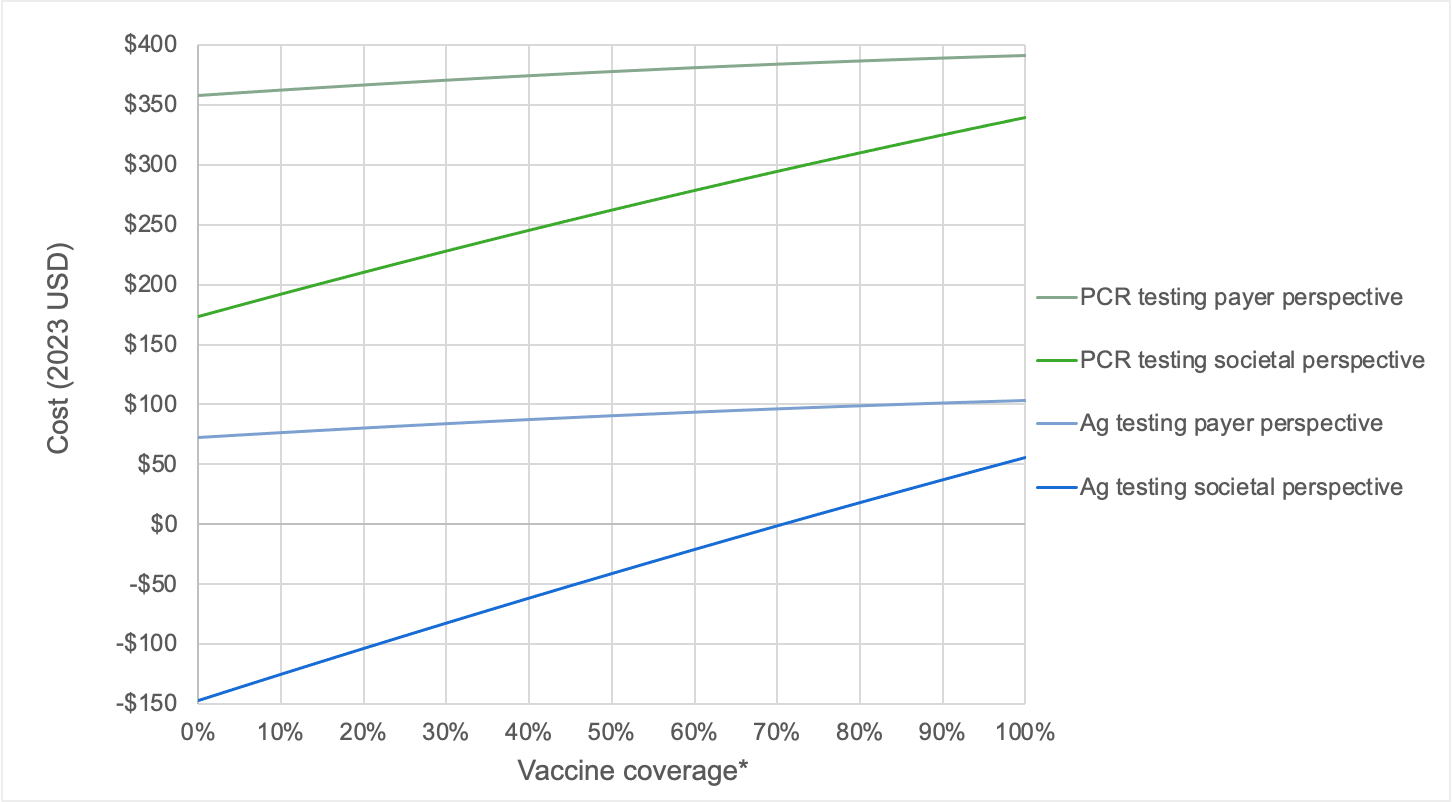
**

*Vaccine coverage represents proportion of the population with at least one dose of COVID-19 vaccine

### **Appendix Figure 3.c.** Quality-adjusted life years per adult shelter resident by vaccine coverage, test type, and perspective: Ag testing vs. no surveillance, PCR testing vs. Ag testing


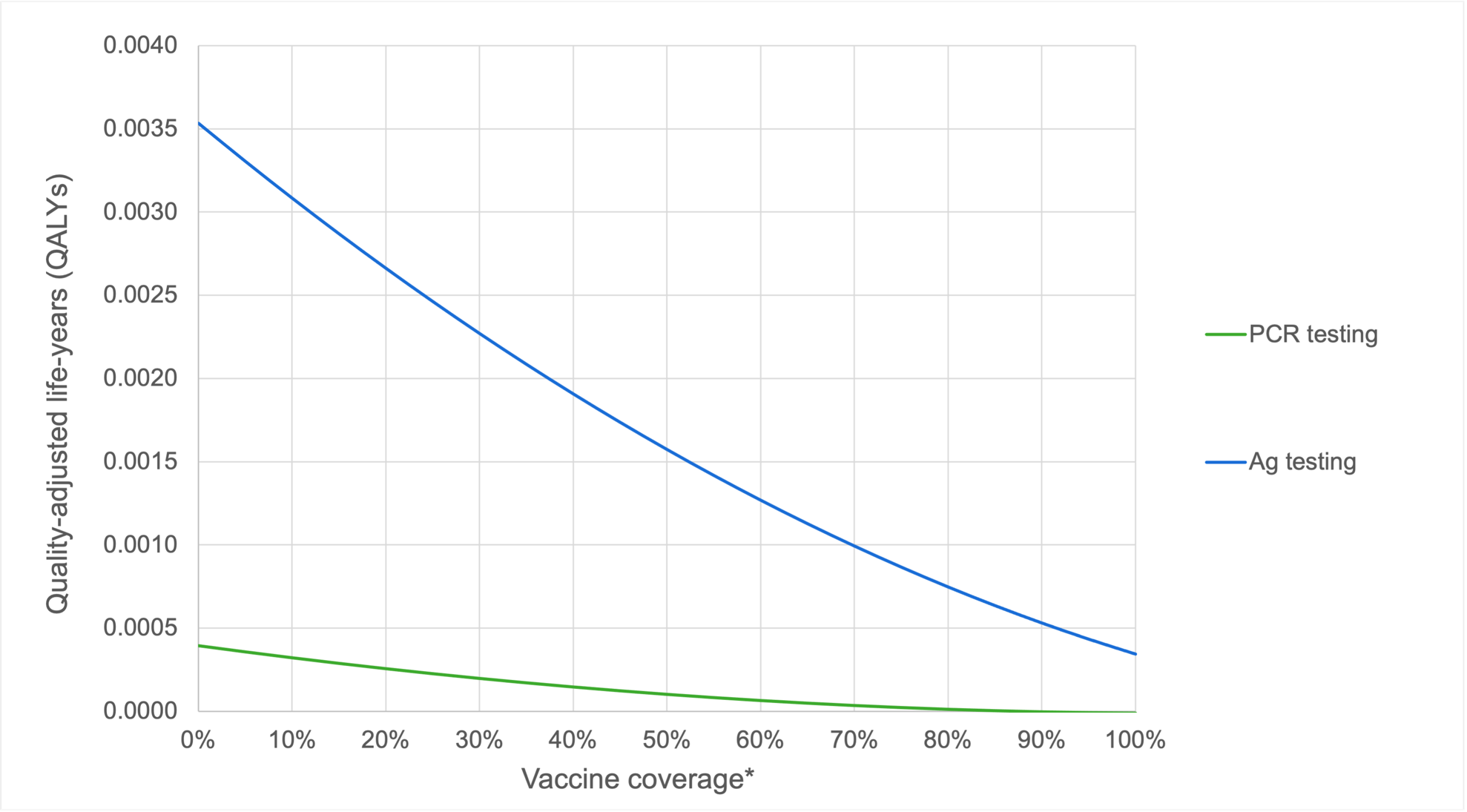
*Vaccine coverage represents proportion of the population with at least one dose of COVID-19 vaccine

### **Appendix Figure 3.d.** Quality-adjusted life years per adult shelter resident by vaccine coverage, test type, and perspective: Ag testing vs. no surveillance, PCR testing vs. no surveillance

**
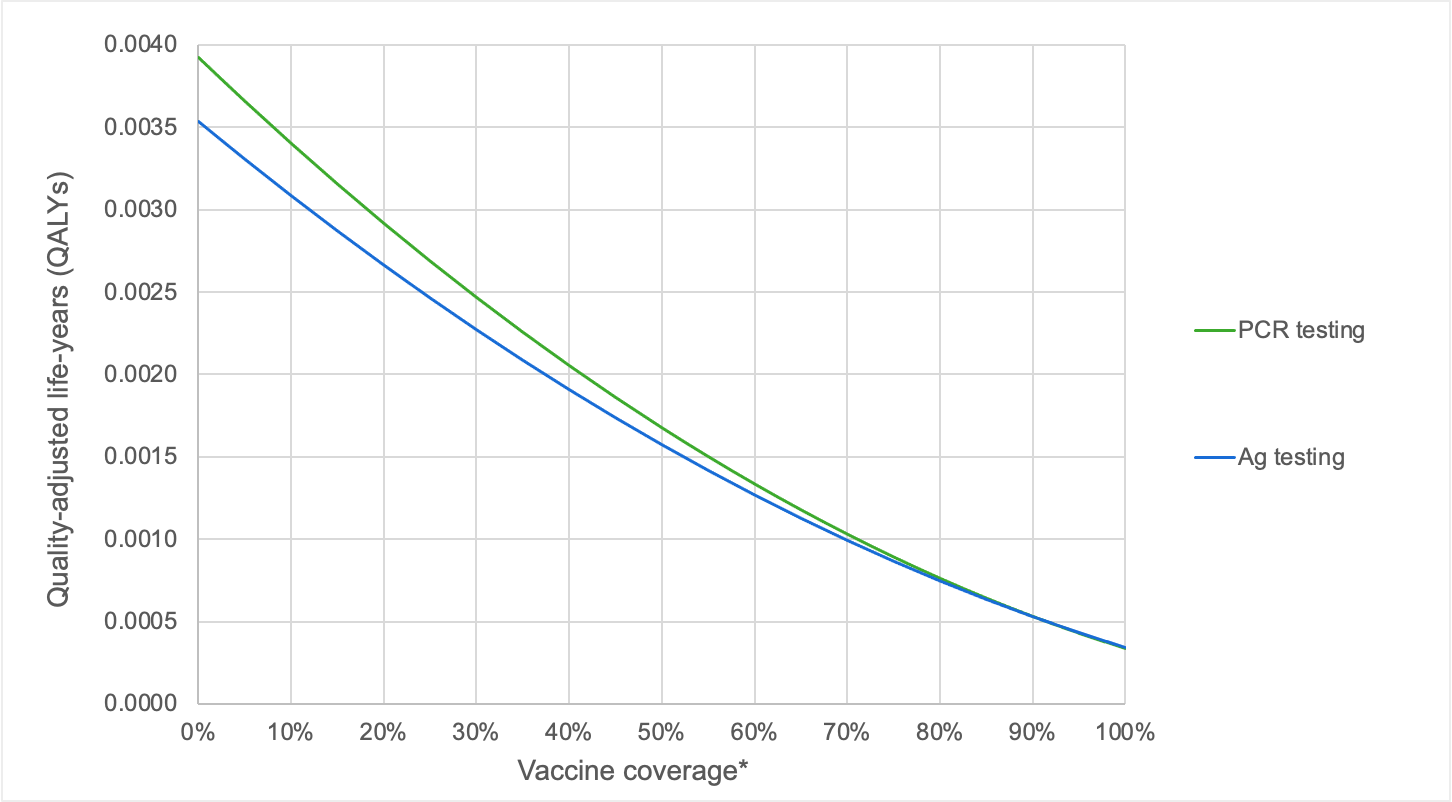
***Vaccine coverage represents proportion of the population with at least one dose of COVID-19 vaccine

#### **Appendix Figure 4.a-d.** Tornado Diagram: one-way sensitivity analyses on the incremental cost-effectiveness ratios (ICERs) of polymerase chain reaction (PCR) testing and antigen (Ag) testing compared to no surveillance by perspective

| **Healthcare payer perspective** |
| --- |
| **a. *Ag testing vs. no surveillance* 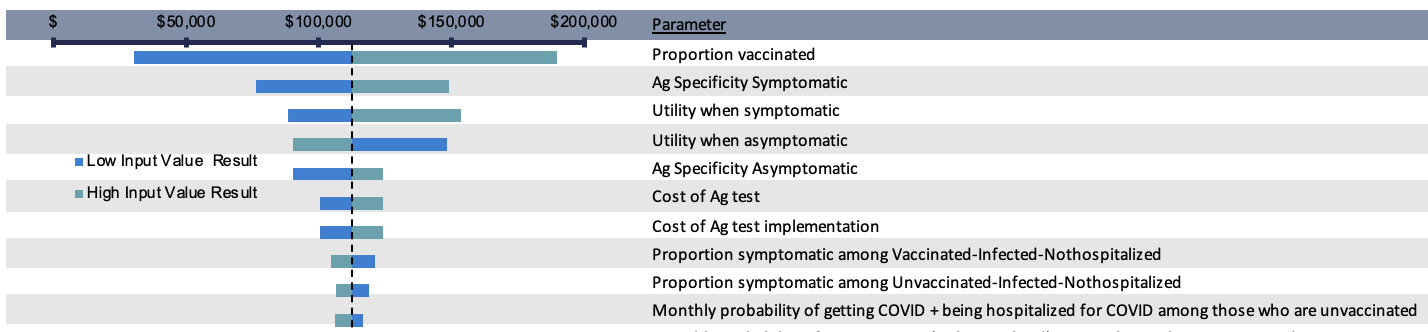** |
| **b. *PCR testing vs. no surveillance***  **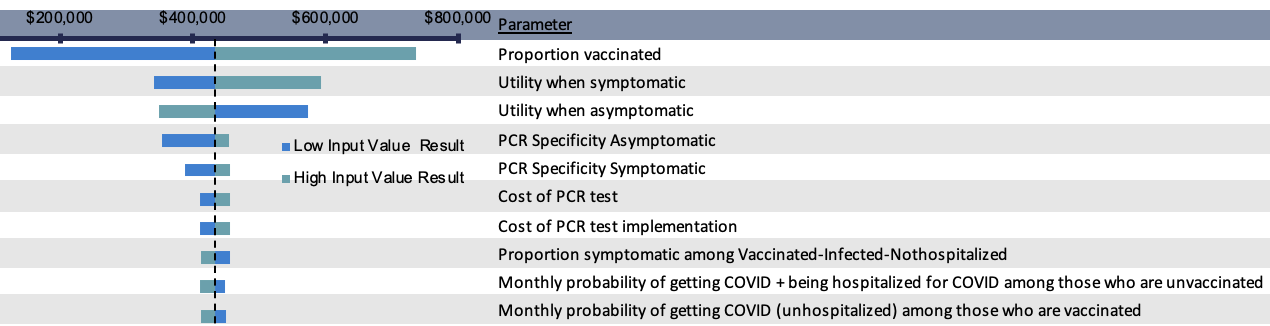** |
| **Limited societal perspective** |
| **c. *Ag testing vs. no surveillance***  **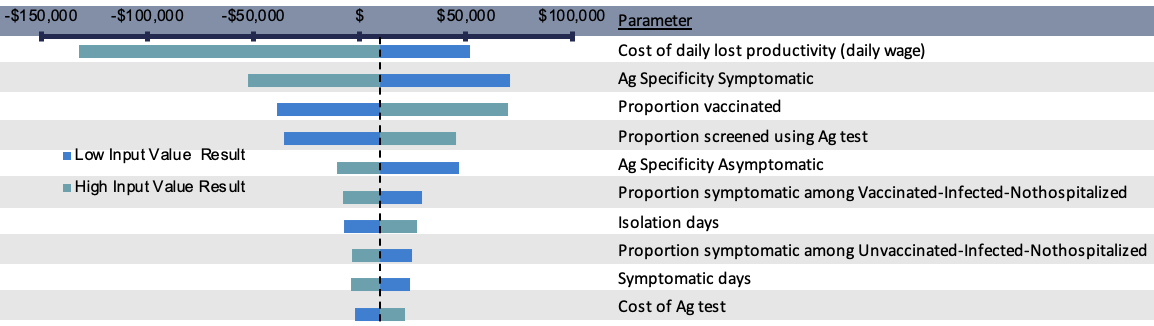** |
| **d. *PCR testing vs. no surveillance***  **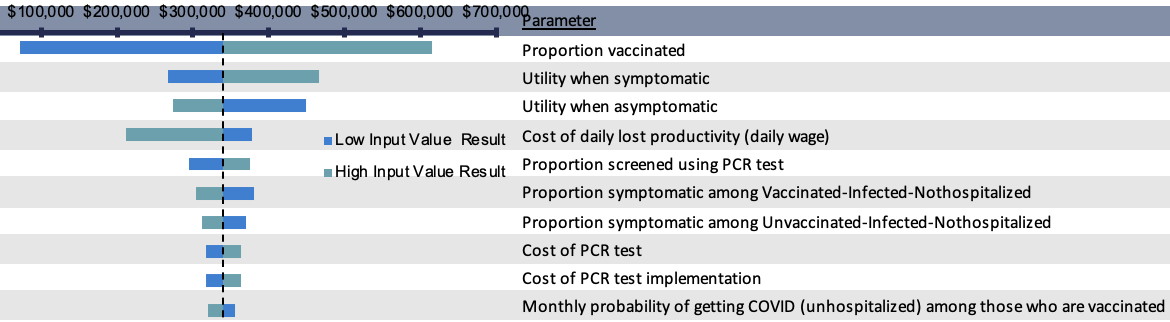** |

#### **Appendix Figure 5.** Cost-effectiveness by proportion of shelter residents screened, test type, and perspective: Ag testing vs. no surveillance testing, PCR testing vs. no surveillance testing


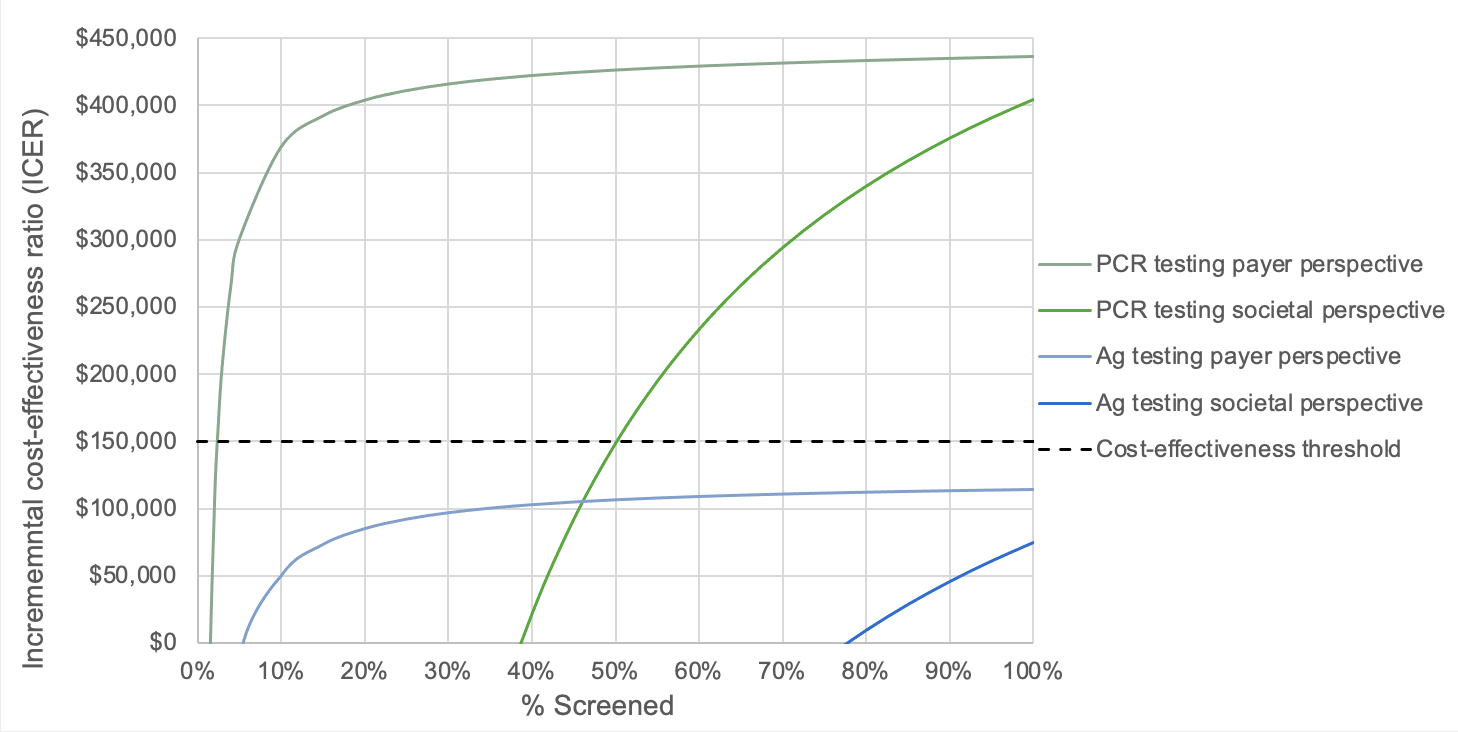


All monthly COVID-19 testing strategies pictured are compared to no surveillance (PCR testing compared to Ag testing was never cost-effective)

### **Appendix Methods.** Description of Seattle Flu Study (SFS)

Our model was parameterized using data from the SFS where possible. The SFS began respiratory virus surveillance of homeless shelter residents in Seattle King County, WA on January 1, 2020. Study enrollment was initially limited to residents with cough alone or ≥2 new or worsening acute respiratory illness signs or symptoms with onset in the past seven days (i.e., fever, cough, sore throat, dyspnea, rhinorrhea, myalgia, or headache), with the exception that asymptomatic residents were eligible to participate 1x/month. In response to the identification of SARS-CoV-2 community transmission in Washington State on February 24, 2020,^11^ eligibility was further expanded to include all 1x/week regardless of symptoms (rather than 1x/month).^12,13^ All shelter residents were encouraged to test monthly.

Study consent was obtained from all persons. After consent was obtained, an enrollment questionnaire was administered collecting information sociodemographics, self-reported chronic conditions, and illness course symptoms. Collected sociodemographic data included shelter site location, birthdate, sex at birth, race and ethnicity, pregnancy status, and current tobacco use status. Self-reported chronic conditions included neurologic disease, cardiovascular disease, asthma, bronchitis, chronic obstructive pulmonary disease, hepatic disease, diabetes mellitus, immunosuppression, and cancer or other conditions not listed. Illness course questions in the survey included self-reported symptoms and illness duration. Questionnaire symptoms included subjective fever, cough, sore throat, dyspnea, rhinorrhea, myalgia, headache, fatigue, sweats, nausea or vomiting, chills, diarrhea, rash, and ear pain or discharge. Loss of taste or smell was added on April 1, 2020.

Survey questionnaires were administered on an electronic tablet at the time of respiratory sample collection and data was stored on Research Electronic Data Capture. A respiratory sample was also collected at enrollment by using midturbinate sterile nylon flocked swabs (Copan Diagnostics, <https://www.copanusa.com>). From July 22, 2020‒November 1, 2020, anterior nares swabs were used due COVID-19 pandemic‒ associated supply changes. Respiratory specimens were initially obtained by study staff. However, the procedure was converted to study staff supervised self-collected swab specimens with the community spread of SARS-CoV-2.

Specimens were stored at 4°C in universal transport medium. Respiratory specimens were tested by using the TaqMan RT-PCR platform (Thermo Fisher OpenArray, https://www.thermofisher.com) that included influenza virus (A, B and C), respiratory syncytial virus (RSV-A and RSV-B), human parainfluenza (HPIV 1-4), human coronaviruses (HCoV- NL63, HCoV-OC43, HCoV-229E, HCoV-HKU1), rhinovirus, enterovirus, human bocavirus, human parechovirus, human metapneumovirus, adenovirus and SARS-CoV-2 (from January 1, 2020 onward). Specimens collected from January 1, 2020, onward were tested for SARS-CoV-2 with those collected after February 25, 2020 tested prospectively. For the purposes of this study, we categorized SARS-CoV-2 inconclusive results as positive (n=22, 0.15%). A cycle threshold (Ct) was generated for each virus-positive sample.

**References.**

1. Savitsky, L. M. & Albright, C. M. Preventing COVID-19 Transmission on Labor and Delivery: A Decision Analysis. *Am J Perinatol* **37**, 1031–1037 (2020).

2. Maya, S. *et al.* Optimal strategies to screen health care workers for COVID-19 in the US: a cost-effectiveness analysis. *Cost Eff Resour Alloc* **20**, 2 (2022).

3. Maya, S. *et al.* COVID-19 Testing Strategies for K-12 Schools in California: A Cost-Effectiveness Analysis. *Int J Environ Res Public Health* **19**, 9371 (2022).

4. Baggett, T. P. *et al.* Clinical Outcomes, Costs, and Cost-effectiveness of Strategies for Adults Experiencing Sheltered Homelessness During the COVID-19 Pandemic. *JAMA Network Open* **3**, e2028195 (2020).

5. Neilan, A. M. *et al.* Clinical Impact, Costs, and Cost-effectiveness of Expanded Severe Acute Respiratory Syndrome Coronavirus 2 Testing in Massachusetts. *Clin Infect Dis* **73**, e2908–e2917 (2021).

6. Losina, E. *et al.* College Campuses and COVID-19 Mitigation: Clinical and Economic Value. *Ann Intern Med* **174**, 472–483 (2021).

7. Paltiel, A. D., Zheng, A. & Walensky, R. P. Assessment of SARS-CoV-2 Screening Strategies to Permit the Safe Reopening of College Campuses in the United States. *JAMA Netw Open* **3**, e2016818 (2020).

8. Paltiel, A. D., Zheng, A. & Sax, P. E. Clinical and Economic Effects of Widespread Rapid Testing to Decrease SARS-CoV-2 Transmission. *Ann Intern Med* **174**, 803–810 (2021).

9. Du, Z. *et al.* Comparative cost-effectiveness of SARS-CoV-2 testing strategies in the USA: a modelling study. *The Lancet Public Health* **6**, e184–e191 (2021).

10. Du, Z. *et al.* Cost-effective proactive testing strategies during COVID-19 mass vaccination: A modelling study. *Lancet Reg Health Am* **8**, 100182 (2022).

11. Chu, H. Y. *et al.* The Seattle Flu Study: a multiarm community-based prospective study protocol for assessing influenza prevalence, transmission and genomic epidemiology. *BMJ Open* **10**, e037295 (2020).

12. Rogers, J. H. *et al.* Characteristics of COVID-19 in Homeless Shelters : A Community-Based Surveillance Study. *Ann Intern Med* **174**, 42–49 (2021).

13. Rogers, J. H. *et al.* Incidence of SARS-CoV-2 infection and associated risk factors among staff and residents at homeless shelters in King County, Washington: an active surveillance study. *Epidemiol Infect* **151**, e129 (2023).
